# Rosetting in *Plasmodium vivax*: A Cytoadhesion Phenotype Associated with Anaemia

**DOI:** 10.1371/journal.pntd.0002155

**Published:** 2013-04-04

**Authors:** Alejandro Marín-Menéndez, Azucena Bardají, Flor E. Martínez-Espinosa, Camila Bôtto-Menezes, Marcus V. Lacerda, Jon Ortiz, Pau Cisteró, Mireia Piqueras, Ingrid Felger, Ivo Müeller, Jaume Ordi, Hernando del Portillo, Clara Menéndez, Mats Wahlgren, Alfredo Mayor

**Affiliations:** 1 Barcelona Centre for International Health Research (CRESIB), Hospital Clinic/Universitat de Barcelona, Barcelona, Spain; 2 Department of Microbiology, Tumor and Cell Biology, Karolinska Institutet, Stockholm, Sweden; 3 Gerência de Malária, Fundação de Medicina Tropical do Amazonas Dr. Heitor Vieira Dourado, Manaus, Brazil; 4 Instituto Leônidas e Maria Deane, Fiocruz Amazônia, Manaus, Brazil; 5 Swiss Tropical and Public Health Institute, Basel, Switzerland; 6 Papua New Guinea Institute of Medical Research, Madang, Papua New Guinea; 7 Department of Pathology, Hospital Clinic, University of Barcelona, Barcelona, Spain; Institute of Tropical Medicine (NEKKEN), Japan

## Abstract

**Background:**

*Plasmodium vivax* can potentially lead to life-threatening episodes but the mechanisms underlying severe disease remain poorly defined. Cytoadhesion of infected erythrocytes may contribute to *P. vivax* sequestration and organ injury although its physiological impact is still unknown. Here, we aimed to describe clinically-relevant cytoadhesive phenotypes of *P. vivax* isolates.

**Methodology/Principal findings:**

Rosetting and adhesion to CSA, CD36, ICAM1, placental and brain cryosections were determined in *P. vivax* peripheral isolates from 12 pregnant women, 24 non-pregnant women and 23 men from Manaus (Brazil). *P. falciparum* co-infection was excluded by PCR and *P. vivax* isolates were genotyped by assessing the size polymorphism of microsatellites ms2, ms20 and msp1F3 through capillary electrophoresis of PCR products. *P. vivax* monoinfection was confirmed by PCR in 59 isolates, with 50 (85%) of them being single-clone infections. One *P. vivax* haplotype was more frequently found among pregnant women (33%) than in non-pregnant women (0%) and men (4%; p = 0.010). Rosetting was observed in 64% of the isolates, adhesion to CSA in 15%, to ICAM1 in 12% and to placental cryosections in 9%, being similar among pregnant and non-pregnant groups. Intensity of rosetting was higher among anaemic individuals compared to non-anaemic (p = 0.010) and decreased with increasing haematocrit (p = 0.033) and haemoglobin levels (p = 0.015).

**Conclusions/Significance:**

*P. vivax* peripheral isolates from pregnant women do not exhibit a prominent adhesion to CSA, although other parasite phenotypes still unknown may increase the propagation of certain *P. vivax* clones observed among pregnant hosts. Rosetting is a frequent cytoadhesive phenotype in *P. vivax* infections that may contribute to the development of anaemia.

## Author Summary

Despite being considered a relatively benign disease, *Plasmodium vivax* infection has been recently associated with fatal outcomes. The mechanisms contributing to severe disease in *P. vivax* malaria remain largely unknown, although scarce evidences suggests that similarly to *P. falciparum*, *P. vivax* may also adhere to host receptors on the vascular endothelium or on uninfected erythrocytes to form ‘rosettes’. Such cytoadhesion phenotypes might contribute to mild sequestration of *P. vivax* and poor clinical outcomes. The present study aimed to describe clinically relevant cytoadhesive phenotypes of *P. vivax* infected erythrocytes isolated from peripheral blood of pregnant and non-pregnant patients in the Brazilian Amazon. We did not observe any specific cytoadhesion phenotype associated to pregnancy, although a *P. vivax* haplotype was more frequent among pregnant women than in non-pregnant hosts. This finding suggests that other parasite phenotypes still unknown may increase the propagation of certain *P. vivax* clones among pregnant hosts. In addition, we found that rosetting was a frequent cytoadhesive phenotype in *P. vivax* infections that was associated with an increased risk of anaemia. This study places cytoadhesion and specifically rosetting as a target for the development of new therapies to treat or prevent life-threatening *P. vivax* malaria.

## Introduction

*Plasmodium vivax* is an important cause of morbidity outside Africa [1] that can induce severe complications, including cerebral malaria, acute respiratory distress and severe anaemia [2], [3], and potentially lead to life-threatening episodes [4]. Although in some of these studies co-infection with *P. falciparum* and/or underlying bacterial or viral infections were not ruled out, these reports have challenged the dominant paradigm of *P. vivax* as a benign infection.

The mechanisms underlying severe disease in *P. vivax* malaria remain poorly defined [5]. Processes central to the development of severity in *P. falciparum* malaria, such as high parasite biomass and reduced deformability of infected and uninfected erythrocytes [6], are not found in *P. vivax* malaria. The lower pyrogenic threshold [7] and greater production of pro-inflammatory cytokines during *P. vivax* infection compared with *P. falciparum* [8] points towards a pathological process linked to cytokine-related inflammation. However, recent studies have suggested that cytoadhesion and sequestration of infected erythrocytes (IE), a key pathogenic process involved in severe *P. falciparum* malaria [6], may also contribute to severe disease in *P. vivax* [9], [10], [11], [12].

Early studies performed in Thailand showed that *P. vivax* was able to form rosettes [13], [14], a cytoadhesive phenotype that has been associated with severe *P. falciparum* malaria in African children [15], [16], [17]. Rosetting has been confirmed in more recent studies in Thailand [9], [18], although information is lacking from other endemic areas. Recent *in vitro* studies from Manaus (Brazil) and Thailand have provided evidence that mature *P. vivax*-IEs can cytoadhere to human lung endothelial cells, Saimiri brain endothelial cells and placental cryosections [11]. *P. vivax* seems to lack the property to bind to CD36 [9], [14], a receptor commonly used by *P. falciparum* [16]. In contrast, adhesion to chondroitin sulphate A (CSA), the receptor for adhesion of *P. falciparum* in the placenta [19], has been shown for *P. vivax* isolates collected from non-pregnant adult patients residing in the Brazilian Amazon [11] and Thailand [9]. Hyaluronic acid (HA) and Intercellular Adhesion Molecule 1 (ICAM1) [20], also used by *P. falciparum* to adhere in the placenta [21] and the brain, respectively, has been suggested to mediate adhesion of *P. vivax* [9], although contradictory results have been reported [11]. Finally, *P. falciparum* transgenic lines expressing *P. vivax* VIR proteins were able to adhere to different endothelial receptors expressed in Chinese Hamster Ovarian cells under static conditions. [22], further indicating a cytoadhesive phenotype in *P. vivax*. However, it is not known if these *P. vivax* adhesion phenotypes have any clinical impact in the infected host.

Understanding the factors that determine *P. vivax*-associated morbidity and severe disease can contribute to develop new tools against malaria. Therefore, the aim of this study was to describe clinically relevant cytoadhesive phenotypes of *P. vivax* IEs isolated from patients in the Brazilian Amazon and to characterize adhesive patterns specific for *P. vivax* parasites infecting pregnant women. The present work shows that rosetting is a frequent cytoadhesive phenotype in *P. vivax* infections that may contribute to the development of anaemia.

## Materials and Methods

### Study site and recruitment of participants

The recruitment of participants was conducted between August and November 2011 at the Fundaçao de Medicina Tropical Dr. Heitor Vieira Dourado (FMT-HVD), in Manaus, capital of the Amazonas State, Brazil. In the area, *Anopheles darlingi* is the major malaria vector and the annual parasite index in 2009 was 11.5 cases/1,000 inhabitants. Patients presenting at the FMT-HVD with fever, chills or headache and a *P. vivax* parasitemia equal or higher than 1+ (300–500 parasites/mm^3^) detected in Giemsa-stained blood thick smears [23] were invited to participate in the study. Clinical and demographic data were collected including age, ethnicity, temperature and, in pregnant women, parity and date of last menstruation. A β-HCG test was carried out to rule out pregnancy in those women who reported not being pregnant or did not know her pregnancy status. Before receiving treatment following national guidelines, 10 mL of blood were collected into EDTA-vacutainer tubes and four drops (50 µL each) were spotted onto filter paper (Whatman, 903 TM) that were kept at 4°C in silica gel. Haemogram was carried out with a Sysmex KX21N (Sysmex Corporation-Roche, Japan). Parasite density was assessed by scoring the number of asexual stage parasites until 500 leukocytes had been counted and number of leukocytes in the haemogram was used to convert parasite numbers observed by microscopy to parasites per µL.

### Ethics statement

Written informed consent was obtained from all participants, and all clinical investigations were conducted according to the principles expressed in the Declaration of Helsinki. The study was approved by the Ethics Committee Board of the FMT-HVD and the Hospital Clinic of Barcelona.

### Parasite isolation and enrichment

Immediately after blood collection, erythrocytes were pelleted by centrifugation, washed three times and re-suspended in RPMI 1640 medium to a haematocrit of 20%. Five mL of this suspension was overlaid on a 2.5-mL 45% Percoll solution in a 15-mL tube and centrifuged at 1500 g during 15 min. The layer with mature IEs was collected, washed twice, re-suspended in RPMI 1640 after two more washes and examined in Giemsa-stained smears [11]. IEs were passed then through a Plasmodipur filter to remove white blood cells, eluted with 40 ml of RPMI 1640 medium and diluted with the remaining erythrocytes of the same patient to a parasitemia of 2.5–5% and a 2.5–5% haematocrit in binding medium (RPMI1640-HEPES, pH 6.8).

### Binding to placental and brain cryosections

Placental and brain biopsies from Spanish donors never exposed to malaria were snap frozen in Tissue-Tek OCT (Sakura, Alphen aan den Rijn, The Netherlands) and stored at −70°C. Fifty µL of the suspension of IEs at 2.5–5% parasitemia and 2.5–5% haematocrit were placed over two 5 µm serial sections mounted onto glass slides and incubated for 1 hour at 37°C in duplicate. The samples were washed 3 times with binding medium and fixed in 2% glutaraldehyde in phosphate buffered saline (PBS) for 2 hours. After air drying and staining with 5% Giemsa for 10 minutes, cryosections were examined by microscopy and IEs counted in 30 high-powered fields and expressed as IEs/mm^2^.

### Binding to purified receptors

Twenty µL of 50 µg/mL of each receptor (CSA, CD36, ICAM1 and bovine serum albumin [BSA] as a negative control) diluted in PBS were placed in individual plastic cover slips, incubated overnight at 4°C in a humid box and blocked with 50 µL of 0.1% BSA in PBS during 45 min at 37°C. After washing, 40 µL of IE suspension were added in triplicate and incubated for 1 hour at 37°C. The samples were softly washed 10 times with adhesion media and the adherent cells fixed with 2% glutaraldehyde in PBS and stained with 5% Giemsa for 10 minutes. Adherent IEs to each receptor were counted by observation of 30 high-power fields and expressed in IEs/mm^2^ previous subtraction of the number of uninfected erythrocytes counted. The CSA-binding *P. falciparum* CS2 strain was used as positive control [24].

### Rosetting assays

Seventy µL of IEs at 2.5–5% parasitemia and 2.5–5% haematocrit were incubated for 1 hour at 37°C in rosetting media (RPMI1640-HEPES supplemented with 10% pooled human serum [Sigma] heat inactivated at 56°C for 30 min). Twenty µL in triplicate were stained with 45 µg/mL of acridine orange and examined by direct light and fluorescence microscopy (Nikon Eclipse 50i, filter 96311 B-2E/C). The proportion of IEs in rosettes was measured after counting 100 IEs in each triplicate, with the adhesion of two or more uninfected erythrocytes to an IE constituting a rosette. Giant erythrocyte rosettes surrounding a cell infected by *P. vivax* were also counted [14]. The *P. falciparum* rosetting strain R29 was used as positive control [25].

### PCR detection and genotyping of *P. vivax*

DNA was extracted from blood onto filter papers using the QIAamp DNA Mini Kit (QIAGEN). Samples were screened for *P. falciparum* and *P. vivax* DNA by a species-specific nested PCR as described elsewhere [26]. A positive and negative-control sample with no template DNA was also run in all reactions. Size polymorphisms for 3 genetic markers (ms2, ms20 and msp1F3) were determined by capillary electrophoresis of PCR products using a 3730×ls DNA analyzer (Applied Biosystems) and analyzed with GeneMarker version 1.6 (Soft-Genetics) [27], [28]. Multiplicity of infection (MOI) was estimated as the highest number of ms2, ms20 or msp1F3 genotypes detected in the sample.

### Definitions and statistical analysis

Gestational age of the pregnant women was calculated based on the date of last menstruation. Thrombocytopenia was considered if platelet count was lower than 150.000/mm^3^ and anaemia if haemoglobin was lower than 11 g/dL. Cytoadhesion data were expressed as the mean of duplicate/triplicate experiments. Cytoadhesion to purified receptors was considered positive if the number of bound IEs/mm^2^ was higher than the mean binding to BSA plus two standard deviations (SD) in at least two of the triplicates and positive for rosetting if a rosette was found in at least two of the triplicates. Binding to cryosections was considered positive if at least one parasite was observed in both duplicates. All data collected were entered into the Excel software (Microsoft Co.) and analysed using Stata version 12.0 software (Stata Corporation).

Fisher's exact test and Mann-Whitney or Kruskal-Wallis test were used to compare categorical and continuous variables, respectively. MOI was compared between groups by ANOVA. Correlations between variables were assessed by Spearman's rank correlation coefficient. Multiple testing correction was performed following Benjamini-Hochberg method. A forward-backward stepwise regression was conducted to select associations between rosetting, anaemia and patient's group (men, pregnant and non-pregnant women) with a significant level for addition to the model of 0.05 and 0.1 for removal. A p-value less than 0.05 was considered as statistically significant.

## Results

Twenty-six men and 39 women were included in the study after being diagnosed with *P. vivax* clinical malaria by microscopy and PCR confirmation of *P. vivax* monoinfection [29]. Median percentage of mature stage IEs after Percoll and Plasmodipur purifications was 89% (Interquartile range [IQR] 77.5–97.0), being only 2% for white blood cells (IQR 0–6). Among these 65 isolates, 59 yielded enough amount of parasite to conduct the adhesion experiments. Twenty-three of the 59 isolates (39%) were collected from men, 24 (41%) from non-pregnant women and 12 (20%) from pregnant women. The three groups were comparable in terms of platelet and white blood cell counts as well as in their parasite densities and prevalence of thrombocytopenia (Table 1). In contrast, median age was higher in men than in women, whereas median haematocrit and haemoglobin levels were lower among pregnant women (Table 1). Prevalence of anaemia was higher among pregnant women than non-pregnant women or men (Table 1).

**Table 1:** Demographic and clinical parameters of participants.

		Women		
	Men	Non-pregnant	Pregnant	
	n = 23	n = 24	n = 12	p
**Age, median (IQR)**	43 (31–52)	32 (24–50)	26 (24–33)	**0.007**
**Haematocrit (%), median (IQR)**	42.7 (40.7–44.3)	37.3 (34.6–41.9)	31.7 (29.6–33.7)	**<0.001**
**Haemoglobin (g/dL), median (IQR)**	13.5 (12.6–13.9)	11.5 (10.9–12.5)	9.8 (9.5–10.4)	**<0.001**
**Anaemia, n (%)**	0 (0)	7 (29)	11 (92)	**<0.001**
**Platelets (10^3^/ml), median (IQR)**	98 (63–136)	83 (58–120)	62 (39–115)	0.778
**Thrombocytopenia, n (%)**	19 (83)	20 (83)	10 (83)	1.000
**WBC (10^3^/µl), median (IQR)**	5.3 (4.4–7.2)	5.4 (4.1–6.1)	5.3 (4.1–6.1)	0.783
**Neutrophils (%), median (IQR)**	66.9 (56.0–76.1)	76.5 (55.4–84.3)	74.5 (63.9–84.2)	0.074
**Lymphocytes (%), median (IQR)**	22.6 (15.0–22.6)	17.7 (8.7–31.3)	15.55 (11.2)	0.305
**Gestational age (weeks), median (IQR)**	-	-	23 (23–28)	NA
**Parity, n (%)**	-	-		NA
**Primigravidae**	-	-	2 (17)	
**Secundigravidae**	-	-	4 (33)	
**Multigravidae**	-	-	6 (50)	
**Parasites/µl (×10^3^), median (IQR)**	3.54 (2.44–8.86)	5.22 (3.46–9.43)	5.63 (0.82–13.39)	1.000
**Single clone infections, n (%)**	20 (87)	20 (83)	10 (83)	1.000
**MOI, mean (SD)**	1.13 (0.33)	1.17 (0.38)	1.17 (0.39)	0.934

P values of the comparison between the three groups (Fisher's exact test and Kruskal-Wallis test for categorical and continuous variables, respectively).

IQR, Interquartile range; WBC, White blood cells; NA, not applicable; MOI, Multiplicity of infection; SD, standard deviation.

Anaemia: <11 g/dL; Thrombocytopenia: <150×10^3^ platelets/mm^3^.

**Figure 1:**
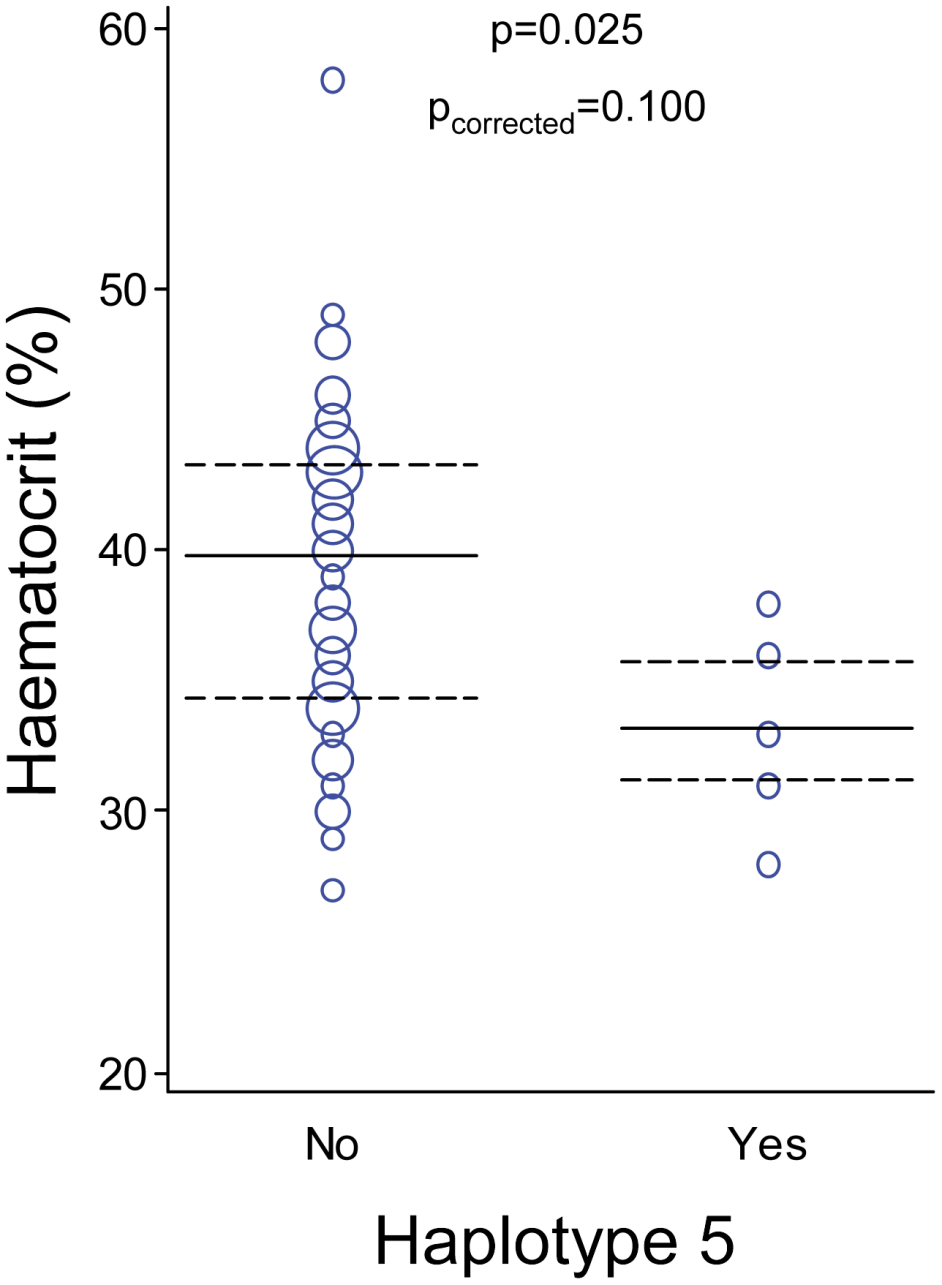
Haematocrit by haplotype 5 of the infecting *P. vivax* isolates. Haplotypes were defined by the size of microsatellites ms2, ms20 and msp1F3 through capillary electrophoresis of PCR products. In the weighted scatter plots, the area of the symbol is proportional to the number of observations. Median values and interquartile ranges are indicated by horizontal continuous and dashed lines, respectively. Differences in haematocrit between individuals infected with haplotype 5 (ms20_194_/ms2_214_/msp1F3_232_) and those infected with other haplotypes were calculated with Mann-Whitney test and multiple testing correction by Benjamini-Hochberg method.

On the basis of the genetic markers analyzed in this study (ms2, ms20, msp1F3), 50 (85%) of the 59 isolates were single-clone infections. Mean MOI was 1.15 (SD = 0.36). Ten msp1F3, 11 ms20 and 12 ms2 alleles were detected (Table S1). Among the 32 unique haplotypes found in the 59 *P. vivax* isolates, 5 were shared by more than 2 isolates (haplotype 1 was observed 12 times [in 20% of samples]; haplotypes 2, 4 and 5 were found 3 [5%], 4 [7%] and 5 [8%] times, respectively). There was no difference between groups neither in the frequency of single-clone infections nor in the MOI (Table 1). Infections consisting on parasites with haplotype 5 tended to be associated with lower haematocrit compared to parasites with other haplotypes (p = 0.025, p_corrected_ = 0.100; Figure 1).

Rosetting was the most frequent cytoadhesion phenotype among the 59 *P. vivax* isolates studied (35/55, 64%), followed by adhesion to CSA (8/53, 15%), ICAM1 (6/52, 12%), placental cryosections (5/58, 9%), CD36 (3/53, 6%) and brain cryosections (1/58, 2%). Fifty isolates had data for all the adhesion experiments, being 27 (54%) of them positive for one of the adhesive phenotypes, 14 (35%) for 2 of the cytoadhesive phenotypes and only 2 (4%) for 3 or more. Among isolates showing binding, cytoadhesion values were counted up to 18.7% for rosetting, 146 IEs/mm^2^ for CSA, 191 IEs/mm^2^ for ICAM-1, 24 IEs/mm^2^ for CD36, 32 IEs/mm^2^ for placental sections and 35 IEs/mm^2^ for brain sections (Table 2). No association was observed between haplotypes and cytoadhesion phenotypes.

**Table 2:** Prevalence of adhesion phenotypes and intensity of binding among isolates showing a positive binding.

	Positive	Intensity
Binding to:	n (%)	Median (IQR)
**Rosetting (n = 55)**	35 (64)	2.7 (1.7–4.7)^b^
**CSA (n = 53)**	8 (15)^a^	25.0 (15.5–95.0)^c^
**ICAM1 (n = 52)**	6 (12)^a^	29.5 (18.0–155.0)^c^
**CD36 (n = 53)**	3 (6)^a^	18.0 (11.0–24.0)^c^
**Placental sections (n = 58)**	5 (9)	4.0 (3.0–5.0)^c^
**Brain sections (n = 57)**	1 (2)	35.0^c^

a , Mean adhesion to BSA was 2.13 IEs per mm^2^ (standard deviation 1.72), giving a threshold of positivity of 5.57 IEs per mm^2^ (mean+ 2 standard deviations);

b , % infected erythrocytes forming rosettes;

c , Infected erythrocytes/mm^2^.

IQR, Interquartile range; CSA, Chondroitin sulphate A; ICAM1; Intercellular Adhesion Molecule 1.

**Figure 2:**
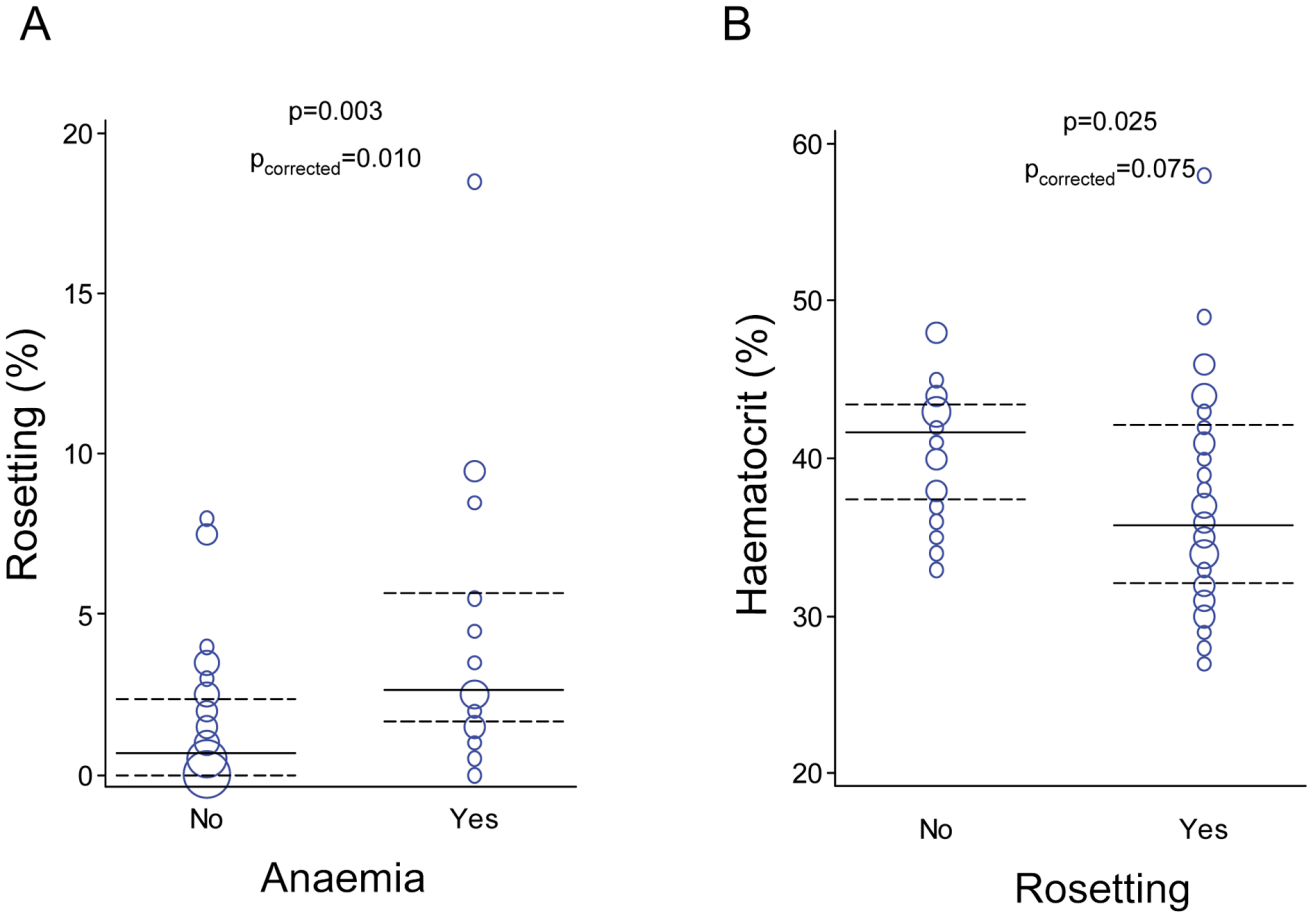
Association between haematocrit and *P. vivax* rosetting. A, Percentage of rosetting by anaemic status of infected individuals. Anaemia (haemoglobin<11 g/dL) was found in 18 of the 59 individuals included. B, Haematocrit of individuals by rosetting phenotype of the infecting *P. vivax* isolate. Rosetting (adhesion of two or more uninfected erythrocytes to an infected erythrocyte) was found in 35 of the 59 *P. vivax* isolates tested. In the weighted scatter plots, the area of the symbol is proportional to the number of observations. Median values and interquartile ranges are indicated by horizontal continuous and dashed lines, respectively. Differences among groups were calculated by Mann-Whitney test with multiple testing correction by Benjamini-Hochberg method.

**Figure 3:**
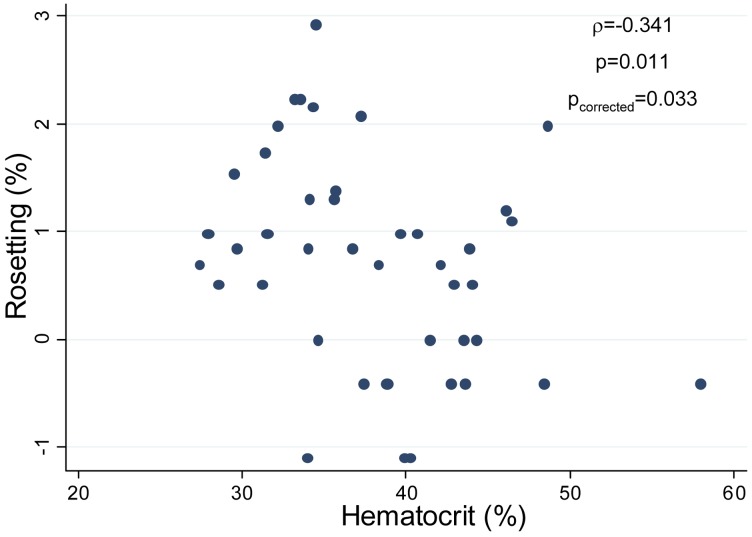
Correlation between haematocrit in infected individuals and intensity of rosetting among *P. vivax* isolates. Correlation between both variables was assessed by Spearman's rank correlation coefficient and multiple testing correction with Benjamini-Hochberg method. The values of p and ρ are illustrated in the graph. The proportion of infected erythrocytes in rosettes (% rosetting) was measured after counting 100 IEs in each triplicate.

Associations with clinical outcomes were assessed for cytoadhesion phenotypes present at frequencies higher than 10% in the isolates studied (rosetting, adhesion to CSA and ICAM1), as well as for the 5 haplotypes detected in the parasite population, with multiple comparisons being accordingly corrected. Frequency of rosetting in parasite isolates from anaemic individuals was higher compared to isolates from non-anaemic individuals (p = 0.003, p_corrected_ = 0.010, Figure 2A). Individuals infected with parasites forming rosettes had a lower haematocrit than individuals with non-rosetting parasites (p = 0.025; p_corrected_ = 0.075, Figure 2B). Similarly, patient's haematocrit decreased with increasing level of rosetting (rho = −0.342, p = 0.011; p_corrected_ = 0.033; Figure 3). The same results were obtained when haemoglobin levels were analysed, being lower among individuals with rosetting parasites (median haemoglobin levels = 11.2 g/dL, IQR [10.1–13.4]) compared to individuals with non-rosetting parasites (12.5 g/dL, IQR [11.9–13.5]; p = 0.020, p_corrected_ = 0.060) and negatively associated with rosetting levels (rho = −0.372, p = 0.005; p_corrected_ = 0.015). The stepwise regression model including anaemia and patient group as covariates selected only anaemia as associated with rosetting, suggesting that the relationship between both variables was not confused by pregnancy status.

Individuals infected with parasites adhering to CSA tended to have infections of higher parasite density (median parasites/µL = 10372, IQR [8242–20303]) than those individuals with non-CSA adhering parasites (median parasites/µL = 4752, IQR [2365–9272]; p = 0.044; p_corrected_ = 0.141). However, no association was found between parasite densities and other adhesion phenotypes tested. Adhesion to CSA was similar in isolates from pregnant and non-pregnant hosts (p = 0.623 for prevalence, p = 0.843 for levels). No association was found between pregnancy and any of the other cytoadhesion phenotypes studied, nor with the ability of parasite isolates to bind to more than one receptor. In contrast, haplotype 5 (ms20_194_/ms2_214_/msp1F3_232_; Supplementary table 1) was more frequent in *P. vivax* isolates from pregnant women (4 out of 12 [33%] than in non-pregnant (0 out of 24 [8%]) and men (1 out of 23 [4%]; p = 0.002; p_corrected_ = 0.010).

## Discussion

This study conducted in an area of moderate *P. vivax* transmission in Brazil shows that *P. vivax* infections with IEs forming rosettes are associated with anaemia, whereas a trend was found between cytoadhesion to CSA and increased parasite density. These adhesive phenotypes may be of clinical relevance and contribute to mild sequestration of *P. vivax* as it has been described in the few autopsy and histological studies performed till now [3], [30], [31]. Importantly, *P. vivax* isolates with a specific parasite haplotype defined by a combination of genetic markers ms20, ms2 and msp1F3 were found at higher frequency among pregnant women, suggesting the existence of particular *P. vivax* strains in this population that may better adapt to pregnant women.

Firstly described for *P. falciparum* in 1988 [32] and thought to interfere with microvascular perfusion in *falciparum* malaria [33], rosetting has been consistently observed in *P. vivax* isolates from Thailand [9], [13], [14], [18]. Our results expand this observation to *P. vivax* isolates from the Brazilian Amazons and suggest that this cytoadhesion phenotype might contribute to anaemia, as it has been observed for *P. falciparum* [16], [34], [35]. Severe anaemia is among the commonest pathologies of severe *vivax* infection [2], [3], [4], [36] and it has been shown to increase the case fatality rate when associated with other signs of severity [4]. The precise mechanism underlying the association between rosetting and anaemia is still unclear. No relationship was found between rosetting and parasite densities, suggesting that other mechanisms different to enhancement of merozoite invasion [37] might be involved, such as destruction of uninfected erythrocytes attached to IEs in rosettes or down regulation of erythrocyte production by abnormal erythrocyte aggregates.

Mature stage *P. vivax* IEs circulating in peripheral blood can adhere *in vitro* to CSA and ICAM1, being the adhesion to CD36 and brain cryosections almost negligible. Cytoadhesion of *P. vivax* observed in this study is less widespread and of lesser magnitude than it has been shown in recent reports [9], [10], possibly due to the use of a more stringent cut-off for positive adhesion based on the background level of unspecific binding to BSA [16]. Intensity of adhesion to CSA is similar to levels found in *P. falciparum* isolates from non-pregnant hosts [38], but much lower than for *P. falciparum* placental isolates [19]. However, the trend towards higher *in vivo* parasite densities among CSA-binding isolates suggests that this cytoadhesion phenotype might eventually contribute to mild parasite sequestration in organs different to the placenta where CSA is present, such as the microvascular endothelium in lungs [39] and brain [38]. However, further studies are needed to explore this association.

*P. vivax* isolates collected from pregnant and non-pregnant hosts did not differ in their prevalence and intensity of adhesion to CSA nor placental sections, suggesting that *P. vivax* isolates from peripheral blood of pregnant women do not exhibit a prominent CSA-adhering phenotype as it is observed for *P. falciparum* [40]. However, pregnant women were found to be more commonly infected by *P. vivax* parasites with a specific haplotype (ms20_194_/ms2_214_/msp1F3_232_) as compared to non-pregnant hosts. This observation suggests that this haplotype might be a surrogate marker for a still unknown adhesion phenotype that may increase the propagation in pregnant women of certain *P. vivax* clones circulating in the population.

This study has several limitations. Firstly, although this is one of the largest data set of *P. vivax* cytoadhesion, 59 *P. vivax* isolates may still be a small number to evaluate associations between adhesion phenotypes, clinical outcomes and pregnancy status. Secondly, the binding assays with trophozoite IEs from peripheral blood may underestimate the cytoadhesive properties of field isolates if mature IEs are effectively sequestered. Also, very low frequency of *P. vivax* placental infection [31] hampered the analysis of parasites directly collected from this organ. Finally, *P. vivax* may bind to other receptors not tested in this study, including hyaluronic acid [9] and others involved in *P. falciparum* severe malaria [41].

In conclusion, this study shows that *P. vivax* isolates exhibit a prominent ability to form rosettes and a less widespread and intense adhesion to purified receptors such a CSA. The association of rosetting with anaemia and the trend observed between adhesion to CSA and increased parasite densities suggest their clinical relevance and potential contribution to mild sequestration observed in *P. vivax* infections [3], [30], [31]. Further studies assessing the cytoadhesion of *P. vivax* ring stages parasites after *in vitro* maturation and of IEs directly collected from placentas of pregnant women, as well as post-mortem studies including immunohistochaemistry and electron microscopy, are needed to determine how *P. vivax* cytoadhesion may contribute to the sequestration of parasites.

## Supporting Information

Supplementary Table S1 Size in pair bases (pb) of ms2, ms20 and msp1F3 in the 59 *P. vivax* isolates by study group. Htc, Haematocrit; MOI, Multiplicity of infection; NA: Not analyzed due to insufficient amount of infected erythrocytes; ND: Not detected.
(DOC)
