# Supplementary material for: Rosetting in Plasmodium vivax: A Cytoadhesion Phenotype Associated with Anaemia
Source: PLoS Negl Trop Dis. 2013 Apr 4;7(4):e2155. doi: 10.1371/journal.pntd.0002155 (PMC3617122; doi:10.1371/journal.pntd.0002155)
Supplement: Supplementary Table S1 — Size in pair bases (pb) of ms2, ms20 and msp1F3 in the 59 P. vivax isolates by study group. Htc, Haematocrit; MOI, Multiplicity of infection; NA: Not analyzed due to insufficient amount of infected erythrocytes; ND: Not detected. (DOC) [file pntd.0002155.s001.doc]

| **Group** | **Htc (%)** | **Rosetting** | **msp1F3 (pb)** | **ms2 (pb)** | **ms20 (pb)** | **Haplotype** | **MOI** |
| --- | --- | --- | --- | --- | --- | --- | --- |
| **Men (n=23)** | | | | | | | |
|  | 42.1 | Yes | 232 | 193 | 197 | 1 | 1 |
|  | 43.8 | Yes | 232 | 193 | 197 | 1 | 1 |
|  | 48.6 | Yes | 232 | 193 | 197 | 1 | 1 |
|  | 40.2 | No | 232 | 193 | 197 | 1 | 1 |
|  | 42.5 | No | 232 | 193 | 197 | 1 | 1 |
|  | 44.3 | No | 232 | 193 | 197 | 1 | 1 |
|  | 33.3 | No | 232 | 202 | 215 | 3 | 1 |
|  | 46.1 | Yes | 232 | 202 | 215 | 3 | 1 |
|  | 38.0 | No | 232 | 214 | 194 | 5 | 1 |
|  | 41.4 | Yes | 232 | 193 | 197 | 6 | 1 |
|  | 43.5 | Yes | 232 | 196 | 200 | 7 | 1 |
|  | 48.4 | No | 232 | 208 | 206 | 8 | 1 |
|  | 39.6 | Yes | 232 | 214 | 206 | 9 | 1 |
|  | 42.9 | Yes | 232 | 229 | 218 | 10 | 1 |
|  | 42.0 | No | 232 | 280 | 212 | 11 | 1 |
|  | 46.4 | Yes | 265 | 202 | 206 | 14 | 1 |
|  | 40.7 | Yes | 274 | 200 | 254 | 16 | 1 |
|  | 42.7 | No | 274 | 205 | 206 | 17 | 1 |
|  | 34.6 | No | 274 | 217 | 206 | 19 | 1 |
|  | 43.3 | No | 274 | 229 | 218 | 21 | 1 |
|  | 44.0 | Yes | 232/268 | 193 | 197 | 27 | 2 |
|  | 48.3 | No | 232/274 | 229 | 218 | 30 | 2 |
|  | 42.0 | NA | 232/274 | 202/229 | 215/251 | 31 | 2 |
| **Non-pregNDnt women (n=24)** | | | | | | | |
|  | 41.3 | No | 232 | 193 | 197 | 1 | 1 |
|  | 37.2 | Yes | 232 | 193 | 197 | 1 | 1 |
|  | 34.3 | Yes | 232 | 193 | 197 | 1 | 1 |
|  | 38.0 | No | 232 | 193 | 197 | 1 | 1 |
|  | 38.8 | Yes | 232 | 193 | 197 | 1 | 1 |
|  | 44.5 | No | 274 | 229 | 218 | 2 | 1 |
|  | 42.7 | NA | 232 | 229 | 203 | 4 | 1 |
|  | 35.6 | No | 232 | 229 | 203 | 4 | 1 |
|  | 32.1 | Yes | 232 | 229 | 203 | 4 | 1 |
|  | 36.7 | No | 232 | 229 | 203 | 4 | 1 |
|  | 57.9 | Yes | 232 | 193 | 197 | 6 | 1 |
|  | 43.6 | No | 232 | ND | 206 | 12 | 1 |
|  | 42.5 | No | 265 | 193 | 197 | 13 | 1 |
|  | 34.5 | Yes | 274 | 208 | 203 | 18 | 1 |
|  | 36.7 | Yes | 274 | 223 | 200 | 20 | 1 |
|  | 31.5 | Yes | 274 | 229 | 251 | 22 | 1 |
|  | 38.3 | Yes | 274 | 229 | 251 | 22 | 1 |
|  | 45.1 | NA | 274 | ND | ND | 23 | 1 |
|  | 34.6 | Yes | 289 | 202 | 197 | 24 | 1 |
|  | 39.9 | No | 298 | 196 | 197 | 25 | 1 |
|  | 37.4 | Yes | 147/247 | 196 | 215 | 26 | 2 |
|  | 35.6 | Yes | 232/274 | 202 | 200 | 28 | 2 |
|  | 34.0 | Yes | 232/274 | 214 | 206 | 29 | 2 |
|  | 27.4 | Yes | 310/253 | 196 | 209 | 32 | 2 |
| **Pregnant women (n=12)** | | | | | | | |
|  | 28.5 | Yes | 232 | 193 | 197 | 1 | 1 |
|  | 33.9 | No | 274 | 229 | 218 | 2 | 1 |
|  | 31.4 | Yes | 232/274 | 193/229 | 218 | 2 | 2 |
|  | 34.1 | Yes | 232 | 202 | 215 | 3 | 1 |
|  | 29.5 | Yes | 232 | 193/202 | 215/197 | 3 | 2 |
|  | 33.2 | Yes | 232 | 214 | 194 | 5 | 1 |
|  | 31.2 | Yes | 232 | 214 | 194 | 5 | 1 |
|  | 35.7 | Yes | 232 | 214 | 194 | 5 | 1 |
|  | 27.9 | Yes | 232 | 214 | 194 | 5 | 1 |
|  | 29.7 | Yes | 232 | 208 | 206 | 8 | 1 |
|  | 33.5 | Yes | 232 | 214 | 206 | 9 | 1 |
|  | 32.0 | NA | 265 | 262 | 254 | 15 | 1 |
